# Correlates of distances traveled to use recreational facilities for physical activity behaviors

**DOI:** 10.1186/1479-5868-3-18

**Published:** 2006-07-19

**Authors:** Gavin R McCormack, Billie Giles-Corti, Max Bulsara, Terri J Pikora

**Affiliations:** 1 School of Population Health, The University of Western Australia, 35 Stirling Highway, Crawley, 6027, Western Australia

## Abstract

**Background:**

Information regarding how far people are willing to travel to use destinations for different types of recreational physical activity behaviors is limited. This study examines the demographic characteristics, neighborhood opportunity and specific-physical activity behaviors associated with distances traveled to destinations used for recreational physical activity.

**Methods:**

A secondary analysis was undertaken of data (n = 1006) from a survey of Western Australian adults. Road network distances between respondents' homes and 1) formal recreational facilities; 2) beaches and rivers; and 3) parks and ovals used for physical activity were determined. Associations between distances to destinations and demographic characteristics, neighborhood opportunity (number of destinations within 1600 meters of household), and physical activity behaviors were examined.

**Results:**

Overall, 56.3% of respondents had used a formal recreational facility, 39.9% a beach or river, and 38.7% a park or oval. The mean distance traveled to all destinations used for physical activity was 5463 ± 5232 meters (m). Distances traveled to formal recreational facilities, beaches and rivers, and parks and ovals differed depending on the physical activity undertaken. Younger adults traveled further than older adults (7311.8 vs. 6012.6 m, p = 0.03) to use beaches and rivers as did residents of socio-economically disadvantaged areas compared with those in advantaged areas (8118.0 vs. 7311.8 m, p = 0.02). Club members traveled further than non-members to use parks and ovals (4156.3 vs. 3351.6 meters, p = 0.02). The type of physical activity undertaken at a destination and number of neighborhood opportunities were also associated with distance traveled for all destination types.

**Conclusion:**

The distances adults travel to a recreational facility depends on the demographic characteristics, destination type, physical activity behavior undertaken at that destination, and number of neighborhood opportunities. Knowing how far adults travel to undertake physical activity will assist in designing supportive neighborhoods and designing future ecological research.

## Background

Land use development patterns contribute to physical inactivity and obesity [1,2]. The increasing distance between households and destinations, a consequence of urban sprawl, results in fewer transport related walking trips as well as reductions in recreational physical activity undertaken at some destinations such as parks and recreational facilities [3,4]. To date, research indicates that physical activity participation is higher in adults who live within close proximity of trails [5,6], parks [7,8], beached [7,9,10], utilitarian destinations such as shops [11] and recreational facilities [12,13]. Fewer studies have examined the relationship between proximity and actual use of recreational destinations [5,14,15].

While proximity to destinations might encourage their use and physical activity participation, other evidence suggests that people are willing to travel outside their local neighborhood to use certain types of recreational facilities. Bull et al[16] found that among Western Australian adults, activities such as aerobics and team sports were mostly undertaken at gyms, health clubs or recreation centers (64.6% and 83.3% respectively). Moreover, many popular destinations such as beaches, used for swimming (33.6%) and running (16.5%) [16], are likely to be located outside an individual's local neighborhood. Other Western Australian data show that among the 51.2% of recreational facilities used, the most popular included passive (i.e., designated space for non-organized sporting activities) (58.9%) and active (i.e., designated space for organized sports) (28.5%) public open space, indoor recreation facilities (23.3%), and aquatic centers (9.4%) [17]. Of those reporting the use of recreational facilities, less than half (45.3%) reported the facility being within walking distance. A Western Australian Government commissioned study of metropolitan beach use, found that 43.8% of those who drove to the beach resided within a 10 kilometer radius [18]. Our own data suggest use of public open space is sensitive to distance from home [13], but that adults are willing to travel further to use other recreational facilities used for vigorous activities and team sport (e.g., beaches, formal recreational facilities).

Post World War II suburban environments have resulted in increased travel distances to destinations. Ross [19] suggests that activities such as shopping, recreational and other activities are generally no longer undertaken in local neighborhoods. This is supported by Western Australian data that shows 24% of leisure and recreational trips are less than 1 kilometer from home, while 30% are between 1 and 5 kilometers [20]. Hence, approximately three-quarters of trips for leisure – including active (e.g., sport and exercise) and passive activities (e.g., spectating and socializing) – appear to be outside the local neighborhood. Another descriptive study involving Perth residents found that people traveled further than their local area to use indoor sporting and tennis facilities, however, more locally available swimming pools, parks, and squash courts were used [21]. Furthermore, some evidence suggests travel distances may vary according to the type of activity, rather than the type of facility. For example, recreational boaters, sailors and water-skiers were less willing to travel as far as pleasure cruisers or fisherman [22].

Econometric models of travel behavior based on the derived travel demand framework hypothesize trip making (i.e., travel distance, time, and mode) as a function of money or time cost of travel, individual income, socio-demographic characteristics and built environmental characteristics [23]. Hence, distances traveled reflect locational as well as demographic and behavioral characteristics [24,25]. Hanson and Hanson [25] found that men visited recreational destinations that were on average 1.3 kilometers further away than those visited by women. Bagley and Moktarian [24], not considering the destination type used, reported that men traveled further compared with women regardless of mode (walking/cycling, transit, and vehicle). Nevertheless, a limitation of studies to date is the broad range of destinations defined as recreational. In studies of travel behavior, leisure or recreational activities have generally included both passive (e.g., socializing, cinema, attending sporting events) and active (e.g., sport, exercise) forms of activity.

The spatial location of opportunities in the local neighborhood may also affect travel behavior as well as the activity undertaken at the destination [26]. The spatial distribution of destinations affects both accessibility and mobility [27]. Handy [28] found that areas with higher levels of local accessibility resulted in shorter trip lengths. Lund [29] found that the frequency of utilitarian walking trips was higher among residence of neighborhoods with access to parks and retail. Furthermore, higher levels of accessibility and local opportunity measured through cumulative or gravity functions have been associated with higher levels of physical activity independent of demographic characteristics [7,12,13,15]. Having limited opportunities available in their immediate areas may force people to travel outside their neighborhoods.

Understanding more about how the proximity of recreational destinations affects physical activity is important because limited access to facilities has been identified as a barrier to physical activity participation [30]. However, the relationship is complex affected by both the location and the activity undertaken at that location [31]. The desire to undertake an activity combined with demographic attributes of the traveler might be sufficient to overcome distance such that an individual is prepared to use destinations outside their local neighborhood.

Information about how far people are willing to travel to use destinations for different types of physical activity behaviors, and whether demographic characteristics are associated with travel distance to specific recreational facilities is limited. Thus, using an econometric model of travel behavior the objective of this descriptive study was to examine whether demographic characteristics, neighborhood opportunity and specific-physical activity behaviors or purpose (i.e., walking, vigorous-intensity, and moderate-intensity physical activity) were associated with distances traveled to destinations used to undertake recreational physical activity in Perth, Western Australia.

## Methods

### Sample design

This study involved a secondary analysis of a sub-sample of data collected from the Study of Environmental and Individual Determinants of Physical activity (SEID 1). The SEID 1 study was initially undertaken to examine the relationship between social disadvantage, the physical environment and recreational physical activity and included a cross-section of adults aged 18 to 59 years (n = 1803, response rate 52.9%) from a 408 km^2 ^area of Perth, Western Australia [13]. Subjects were recruited from 277 census collectors districts (CCD) from the top (137 CCDs and n = 929) and bottom (140 CCDs and n = 874) percentiles of social disadvantage. Data collection was conducted in late spring 1995 and the procedure involved a face-to-face interview in the respondent's home. Further details of the respondent recruitment have been explained elsewhere [13]. The University of Western Australia Human Rights Committee granted ethics approval.

### Data collected

During interviews, respondents reported type, frequency, duration and destinations used for different types of physical activity over the past two weeks. Physical activities included walking for recreation, walking for transport (i.e., to get to or from destinations including work, shops or public transport), moderate-intensity, and vigorous-intensity physical activity (i.e., activities that made them breath hard or puff and pant). The reliability and validity of these items have been demonstrated in Australian contexts [32]. Demographic data were also collected. Respondent residential location and the spatial location of all recreational destinations within the study area, including those reported being used for physical activity, were geocoded using MapInfo. ArcInfo Geographical Information Systems software was used to undertake road network analysis to determine the shortest road network distance in meters between respondent's homes and all destinations. Only those respondents reporting the use of a destination to participate in physical activity for recreation were considered for this study (n = 1,006). The destinations in this study excluded streets and the homes.

### Independent variables

Physical activities undertaken at recreational destinations were aggregated into five categories: 1) walking only; 2) vigorous-intensity only; 3) moderate-intensity only; 4) other recreational activity only, and; 5) multiple recreational activities. 'Other recreational activity' included other physical activities undertaken excluding walking, jogging/running, swimming, aerobics, team sports, tennis/squash, and cycling which were captured by the walking, vigorous-intensity and moderate-intensity categories. 'Multiple recreation activities' was used to identify those destinations at which more than one physical activity was undertaken (e.g., swimming and walking at the same beach). Physical activity in this case is akin to trip purpose used in travel behavior models [23] (e.g., purpose of using formal recreation facility was for vigorous activity only). The original purpose of the data collection was not to examine travel behavior [13] hence a measure of travel cost was not collected.

Demographic characteristics including gender, age (i.e., 18 to 34, 35 to 44, and ≥ 45 years) annual household income (i.e., <$34,999, $35,000 to $59,999, ≥ 60,000, and refused/don't know), and club affiliation (member, and not a member) were included in the current analysis. In addition, social disadvantage, a Socio-Economic Index for Areas (SEIFA) calculated by the Australian Bureau of Statistics, was used to categorize respondents as either socio-economically advantaged (i.e., top percentile) or disadvantaged (i.e., bottom percentile). The index reflects income, occupation, education, and employment at the census collect district level.

A neighborhood cumulative opportunity variable representing the density or count of destinations located within a respondent's local neighborhood was calculated. Measures of cumulative opportunity represent the number of opportunities – in this paper the number of recreational destinations – that can be reached within a given travel distance or time however, a criterion or 'gold standard' distance or time has not been established [27]. Our measure of neighborhood opportunity included destinations within a 1600 meter road network distance of the respondent's home – corresponding to an upper limit of destinations that could be walked to in 15 minutes or driven to in less than 5 minutes [33]. Each respondent had three separate measures of neighborhood opportunity, one for each destination type: beach and rivers; parks and ovals; and formal recreational facilities. Because access to a motor vehicle may potentially increase the number of recreational opportunities available to an individual, a mobility variable with two categories representing access to a motor vehicle (i.e., always, and none to sometimes) was also included.

### Dependent variables

Destinations used for physical activity were grouped into three main types: 1) beaches and rivers; 2) parks and ovals, and; 3) formal facilities (e.g., health clubs, recreation/leisure centers, community swimming pools, local halls, gymnasiums, sports stadiums etc). The outcome variables used in the analyses road network distances, in meters (m), to each of the three destination types. Skewed distributions of the distances to the three destination types resulted in log transformations of distance.

### Statistical analyses

The analysis was stratified by destination type. Three separate models were specified to examine the association between demographic characteristics, neighborhood opportunity, and physical activity behaviors and 1) distance to a used beach or river; 2) distance to a used park or oval, and; 3) distance to a used formal recreation facility. The dataset included a multiple response structure (n = 1606 observations from n = 1006 respondents). Inspection of the sample size after stratification by destination type showed the following breakdown: n = 453 observations from 401 respondents within 174 CCDs for use of beaches and rivers; n = 430 observations from 389 respondents within 191 CCDs for use of parks and ovals, and; n = 723 observations from 566 respondents within 233 CCDs for use of formal recreational facilities. Respondents could report undertaking physical activity at several similar destination types (e.g., running at two different beaches). Of those reporting the use of a park or oval, 90.2% did so at a single location during the previous two weeks (i.e., 9.8% of respondents had used multiple parks and ovals). Similarly, of those using formal recreational facilities, 76.7% used only a single formal recreational facility in the previous two weeks, and 88.0% of beach or river users used a single location.

Although the data represented a possible three level hierarchical structure, a two level model was used, i.e., observations (level 1) within CCDs (level 2). This model was used due to the limited number of multiple responses obtained for each person (beaches and rivers mean = 1.12 ± 0.37, range = 1 to 4; parks and ovals mean = 1.11 ± 0.33, range 1 to 3; formal recreational facilities mean = 1.28 ± 0.55, range 1 to 4), which caused problems in estimating variance at the respondent level. There was a higher number of responses within CCDs which lead to the specification of the two level model (beaches or rivers mean = 2.59 ± 2.14, range 1 to 15; parks and ovals mean = 2.24 ± 1.75, range 1 to 13; formal recreational facilities mean 3.10 ± 2.15, range 1 to 13). The use of a two level model was further supported by the level of clustering of observations within CCDs shown after applying an unconditional means model to the logarithm (log) distance i.e., intraclass correlations for formal facilities = 0.16; beach and rivers = 0.43, and: parks and ovals = 0.31. To account for clustering, log distance to destinations was compared between levels of the independent variables using generalized linear mixed model using the PROC MIXED procedure in SAS [34].

Statistically significant differences in log distance within categories of the independent variables were tested using the F-test statistic (type III). For independent variables showing statistical significance (p < 0.05), post hoc (Least Significant Difference) pairwise comparisons were undertaken. For interpretation purposes, the tables and text of the results section present the non-transformed adjusted mean distances together with p-values based on the adjusted log transformed distances.

## Results

Overall, 566 (56.3%) respondents reported using formal recreational facilities, 401 (39.9%) respondents used a beach or river, and 389 (38.7%) respondents used a park or oval. Table 1 presents the frequency of respondents by demographic and physical activity characteristics using formal recreational facilities, beaches and rivers, and parks and ovals. Note that the percentages in Table 1 do not equal 100% as respondents could report using more than one recreational destination type. For example, among men (n = 341), 54.3% used a formal recreational facility, 44.9% used a beach or river, and 37.2% used a park or oval.

**Table 1:** Frequency^1^using formal recreational facilities, beaches and rivers, and parks and ovals by demographic and physical activity characteristics.

**Variable**	**n**	**Formal facilities (%)**	**Beaches and rivers (%)**	**Parks and ovals (%)**
**Gender**				
Male	341	54.3	44.9	37.2
Female	664	57.4	37.3	39.3
				
**Age in years**				
18–34	423	61.5	41.4	36.2
35–44	304	58.6	36.8	37.5
≥ 45	279	45.9	39.1	43.7
				
**Annual income**				
<$34,999	267	50.9	39.3	43.1
$35–59,999	246	59.8	38.2	38.2
≥$60,000	355	60.6	42.0	34.6
Refused/unknown	137	49.6	38.0	41.6
				
**Area level SES**				
Advantaged	586	56.7	42.5	35.8
Disadvantage	420	55.7	36.2	42.6
				
**Club member**				
No	537	37.6	44.9	46.7
Yes	471	77.5	34.2	29.5
				
**Motor vehicle access**				
Sometimes or never	170	51.8	34.7	40.0
Always	830	57.2	41.1	38.3
				
**Destination use**				
Vigorous exercise	381	76.4	18.4	14.7
Walking	363	0	51.8	59.0
Moderate exercise	173	64.7	35.3	5.8
Other	286	76.9	6.3	20.6
Multiple use	138	9.4	53.6	36.2

^1 ^Missing responses removed from percentage calculation. Percentages include rounding.

^2^Neighborhood opportunity represents the count of specific destinations (either formal or beaches/rivers, or parks/ovals) within 1600 meters of a respondent's household (defined as "neighborhood"). Row percentages do not equal 100% as respondents could report using more than one type of recreational destination.

The use of formal recreational destinations was more popular among respondents aged 18–34 years (61.5%) compared with those ≥ 45 years (45.9%) however, the reverse was true for parks or ovals (≥ 45 years 43.7%; 18–34 year olds 36.2%). The most frequently used recreational destination used for physical activity by club members was formal recreational facilities (77.5%) for physical activity, which were the least used destination (37.5%) for non-club members. Compared with other facilities, formal recreational destinations were more frequently used by respondents undertaking vigorous (76.4%), moderate (64.7%), and other (76.9%) physical activity. Beaches and rivers, and parks and ovals were popular among walkers (51.8% and 59% respectively).

The mean, and standard deviation (minimum and maximum shown in parenthesis) number of formal recreational opportunities within respondents' neighborhoods was 2.47 ± 1.69 (0 and 8), beach and river recreational opportunities was 0.65 ± 1.24 (0 and 6), and park and oval opportunities was 4.93 ± 2.33 (0 and 13). The non-transformed mean network distance to all destinations used for physical activity, was 5463.6 ± 5132.0 m.

### Formal recreational facilities used for physical activity

Estimates from the generalized linear mixed models in Table 2 show that after controlling for other independent variables distance traveled to formal recreational facilities differed between the types of physical activity for which the destination was used (p < 0.01). Pairwise comparisons revealed statistically significant (p < 0.05) differences in distances between home and destinations for respondents undertaking other (6796.5 m) compared with vigorous (4922.8 m) and multiple (4097.3 m) physical activities. Respondents undertaking moderate activity traveled further to use formal recreational facilities (6324.4 m) compared with those undertaking vigorous (4922.8 m) and multiple (4097.3 m) physical activities. Although only approaching statistical significance, respondents not belonging to a club used formal recreational facilities that were further away from home compared with club members (6053.4 m vs. 5017.0 m, p = 0.06). Furthermore, for each additional formal recreational facility located in a respondent's neighborhood, the distance reduced by 494.7 m (p < 0.01) between the respondent's household and the formal recreational facility used for physical activity.

**Table 2:** Adjusted mean network distances to visited formal recreation facilities, beach and rivers, and parks and ovals by demographic, accessibility, and physical activity characteristics.

	**Formal facilities**		**Beach and rivers**		**Parks and ovals**	
**Variable**	**Estimate**	**p-value**^1^	**Estimate**	**p-value**	**Estimate**	**p-value**
**Gender**						
Male	5505.5	0.63	6561.3	0.81	3901.4	0.95
Female	5565.0		6819.8		3606.6	
						
**Age in years**						
18–34	5668.5	0.77	7311.8	0.03	4061.5	0.25
35–44	5543.8		6747.3		3397.6	
≥45	5393.4		6012.6		3802.8	
						
**Annual income**						
<$34,999	6074.8	0.78	6964.2	0.18	3781.1	0.62
$35–59,999	5250.1		7297.7		4121.3	
≥$60,000	5644.2		6366.1		4125.4	
Refused/unknown	5171.8		6134.3		2988.2	
						
**Area level SES**						
Advantaged	6170.4	0.13	7311.8	0.02	3645.0	0.37
Disadvantage	4900.1		8118.0		3863.0	
						
**Club member**						
No	6053.5	0.06	6588.0	0.53	3351.6	0.02
Yes	5017.0		6793.1		4156.3	
						
**Motor vehicle access**						
Sometimes or never	5103.6	0.12	6405.1	0.16	3832.9	0.91
Always	5966.9		6976.1		3675.1	
						
**Destination use**						
Vigorous exercise	4922.8	<0.01	7471.3	0.03	4449.0	0.02
Walking	.		5791.0		3333.4	
Moderate exercise	6324.3		7044.1		2021.7	
Other	6796.5		7117.4		4883.5	
Multiple use	4097.3		6028.9		4082.4	
						
**Neighborhood opportunity**^2^	-494.7	<0.01	-1289.8	<0.01	-430.3	<0.01

^1^Based on F-tests (Type III) undertaken on log-transformed distances.

^2^Neighborhood opportunity represents the count of specific destinations (either formal or beaches/rivers, or parks/ovals) within 1600 meters of a respondent's household (defined as "neighborhood").

### Beach and rivers used for physical activity

Distance traveled to use beaches and rivers for recreational activity was associated with age (p = 0.03), area level SES (p = 0.02), and physical activity purpose (p = 0.03) (Table 2). Pairwise comparisons showed that those aged 18 to 34 years used beach and river destinations that were further from home compared with adults aged 45 years and above (7311.8 m vs. 6012.6, p = 0.01). Furthermore, those who used the beach or river for vigorous physical activity traveled further than those using the beach or river for walking (7471.3 m vs. 5791.0 m, p < 0.01) or for multiple activities (7471.3 m vs. 6028.9 m, p = 0.05). Respondents who used beaches and rivers for moderate activity also traveled further than those using beaches and rivers for walking (7044.1 m vs. 5791.0 m, p = 0.03). As disadvantaged areas in Perth are generally located inland, residents of disadvantaged areas traveled further from home to use beaches and rivers compared with respondents living in advantaged areas (8118.0 m vs. 7311.8 m, p = 0.02).

### Parks and ovals used for physical activity

Those who were club members traveled further from home to use parks and ovals for physical activity than non-members (4156.3 m vs. 3351.6 m, p = 0.02). This reflects the fact that the distances to parks and ovals used for recreational physical activity differed depending on the type of activities undertaken (p = 0.02). More specifically, those undertaking "other" activities at parks and ovals traveled further than walkers (4883.5 m vs. 3333.4 m, p < 0.01), those undertaking other "moderate" activities (4883.5 vs. 2021.7, p = 0.03) and those undertaking "multiple" activities (4883.5 m vs. 4082.4 m, p = 0.05). For each additional park and oval opportunity located within an individuals neighborhood, travel to use these facilities decreased by 430.3 m.

## Discussion

This study examined factors associated with the distances people travel to use destinations for physical activity. We found that the physical activity behavior undertaken at a destination was consistently associated with the distances respondents were prepared to travel, regardless of the type of facility i.e., beaches and rivers, parks and ovals, or formal recreational facilities. Those who participated in vigorous physical activity generally traveled further than non-vigorous exercisers to use the same type of destination (i.e., to use parks and ovals, and beaches and rivers). Moreover, travel distances decreased as a function of the number of destinations available within the respondent's neighborhood, regardless of the type of destination examined. Younger adults, those with a higher income and those from socio-economically disadvantaged areas also tended to travel further to use recreational physical activity destinations.

Travel to, and the use of, destinations is an antecedent to physical activity participation because most physical activities are undertaken in places outside the home [16]. However, the purpose for using a destination (i.e., type of physical activity behavior) may also influence the distance individuals are willing to travel. We found that the type physical activity behavior undertaken had a differential influence on the distances traveled to the same type of destination. This suggests that the purpose for which a recreational destination is used (i.e., for walking, vigorous, moderate, other activity and multiple activities) may influence preparedness to travel, more so than the type of destination. For example, in this study, respondents traveled further to use beaches and rivers and parks or ovals for vigorous activity than they did for walking. Noteworthy, is that distances traveled to beaches and rivers, and parks and ovals for walking are further than what is considered a walkable distance [33]. While these destinations are used for recreational walking, modes of transport other than walking are likely to have been used to reach destinations further away. These results are consistent with an early recreational study, which found that the type of recreational behavior influenced distances traveled to the same destination types [22].

From a physical environment point of view, certain destinations may be more attractive for specific types of activity[35], and similar types of destinations (i.e., all parks, all beaches, and all formal recreational facilities) may not be equally appealing for different types of physical activities [22]. For example, similar types of destinations may differ in terms of their facilities and the number and quality of the attributes that make them attractive to users [8]. They may therefore attract different user groups. Our results suggest that individuals may seek out specific destinations, with attributes (e.g., parks for team sports, parks for running or cycling) that match their preferred type of physical activity

Although not reported, respondents who used destinations in this study had many destinations closer to their home than those they actually used: parks or ovals (mean = 2797 ± 2866 m), beaches (mean = 5139 ± 4432 m), rivers (mean = 2254 ± 1875 m), and formal recreational destinations (mean = 2448 ± 1696 m). Urban sprawl and the segregation of land-uses have contributed to greater distances between homes and recreational destinations [4,19] and thus, increased travel distances. However, because of personal preferences, individuals may access recreational destinations further from home. Preferences may vary for certain activities or facilities, knowledge of alternative destinations, constraining factors, or because destinations are accessed while undertaking other errands (e.g., on the way to or from work) [36]. This finding is similar to those of Sallis et al[12] who found that many people perceived less proximate destinations to be more convenient.

The types of local recreational destinations offered may also restrict the choice of physical activity options available to an individual. In Perth, regional and district parks include a wider range of facilities (i.e., include field markings, goal posts, larger play areas, long continuous paths) catering to a broader range of physical activities, including those that are vigorous (i.e., team sports, cycling, running). Neighborhood parks, on the other hand, are smaller and have fewer facilities (i.e., playgrounds) – catering to fewer types of physical activity (i.e., walking, children's play). Moreover, local neighborhood parks and ovals generally only attract users from local surrounding areas [37,38], whereas regional and district parks and ovals attract patrons from a larger hinterland.

The distribution of destinations is important for encouraging and maintaining behavior and frequency of use [26]. Our study showed that the greater the density of recreational destinations located within 1600 m of a respondent's home the shorter the distances traveled to use a recreational destination. Consistent with other travel behavior research [28], this suggests that the more destination options available, the more likely at least one will be used. For example, higher levels of commercial accessibility are associated with shorter trips to shopping destinations [28]. Thus, it is possible that good access to a mix of nearby recreational opportunities, raises awareness about recreational opportunities, increases the likelihood of using and decreases the travel time needed to reach destinations[39] – providing more time to achieve higher levels of physical activity participation.

Contrary to expectations, compared with non-club members, club members used formal recreational facilities that were closer to home, but used parks and ovals that were located farther away. Members of recreational facilities such as gymnasiums or health clubs may be regular users of these facilities and may join conveniently located facilities closer to home. However, club members that were team sport players need to travel further to parks and oval used for fixtures that might vary weekly. The regional nature of formal and informal recreational facilities that cater for team sports such as large multi-purpose sports centers and ovals ensures that there is a greater spatial spread across communities. This finding is supported by Lobo [21] who reported that people travel further than their local neighborhoods to use indoor sports and tennis facilities. Moreover, as our group reported previously [13] use of some facilities (e.g., sporting and recreational centers, and gyms/health clubs and exercise centers) appears to be less sensitive to distance than use of other types of facilities such as public open space, rivers, tennis courts and beaches.

Our study found that distances traveled from respondent's homes to beaches and rivers decreased with advancing age. Older adults generally travel shorter distances and make fewer trips compared with younger adults [40]. One explanation for our finding is that physical activities undertaken by younger adults at beaches and rivers differ to those activities undertaken by older adults. For example, younger adults are more likely than older adults to undertake vigorous-intensity physical activity [41] and those who do vigorous activity, are more likely to use the beach or river for these activities. Moreover, surfing activities which are popular in Perth, are more prevalent among younger compared with older adults [42] but require surf conditions that only are available at certain beaches (i.e., the closest beach may not provide the best conditions) which increases distances traveled. The desire to socialize at the beach also might explain age-related differences in distances traveled to beaches and rivers for physical activity. Indeed, Hecock [43] found that teenagers and college students were attracted to beaches where there were close social and physical proximity to people of a similar age.

Respondents from disadvantaged areas were less likely to use the beach or river, moreover they traveled considerably further do so compared with those residing in advantaged areas. Previous research has found that the availability of local recreational facilities to be lower among residents of socioeconomic disadvantaged areas [44]. Relative to advantaged areas, disadvantaged areas in the Perth metropolitan area are generally located further from beaches and rivers. Moreover, the distances presented in this paper are likely to underestimate the distances traveled by people from low SES areas because many low socioeconomic areas in Perth are situated outside the inland boundary SEID 1 study area (see Giles-Corti and Donovan [13] for more details). Thus, travel distance, cost, and the time involved are likely to be significant barriers to using a beach or river for physical activity for residents of disadvantaged areas. The prevalence of beach or river use was only slightly lower in respondents living in disadvantaged compared with advantaged areas (36.2% versus 42.5%). However, extensive travel distances to free and natural facilities is likely to reduce frequent use because of the cost and inconvenience [45]. This is important, because overall levels of physical activity appear to be higher among residents residing in coastal compared with inland postal code districts [9].

Issues concerning definitions of how to define "neighborhood" in physical activity research have been raised [33]. Physical activity research undertaken to date has defined neighborhood boundaries as geographical areas of 400–1000 m from respondent's homes representing the distance individuals are likely or willing to walk [15,46,47]. The observed travel distances in our study suggest that a significant proportion of destinations used for physical activity are located outside the local neighborhood – supporting Ross's [19] claim that activities (including recreational activity) are no longer undertaken in local neighborhoods. However, the reasons for using recreational destinations outside the local neighborhood appear to be associated with several factors including the availability of local opportunities, demographic characteristics and the type of physical activity to undertaken at the destinations. The neighborhood was not the main locus for trips to the destinations examined in this study hence, our next step is to examine whether the location and type of destination used influences a person's physical activity behavior.

### Limitations

The spatial distribution of homes, recreational destinations, and demographic groups are likely to differ between cities and hence influence generalizability. Thus, the findings from this study may not be transferable to other spatial settings or physical environments. Also noteworthy is that people may make decisions about whether to use a destination based on information other than proximity [39]. For example, reasons for using a trail have included it being a favorable environment, convenient location, safe place to exercise, paved road, presence of mile markers, and freedom from motorized transportation [48].

This study was limited to examining distance data, however, the quality of recreational destinations might be more important than proximity in some instances [8,39]. The cross-sectional data in this study means that causality cannot be determined. Importantly, our results indicate the purpose (i.e., the physical activity behavior) for using a destination interacts with the type of destination and its attractiveness, as a determinant of travel distances. It is also plausible that the distance people travel to destinations determines the types of behavior undertaken. For example, individuals might combine several activities at a destination, which involved a longer trip (e.g., jogging and swimming at the same beach during the same visit). Despite these limitations the strengths of this paper were the use of an established travel behavior model to examine associations, the inclusion of trip purpose as an independent variable (i.e., physical activity behavior), and the examination of distances traveled to specific types of recreational destinations.

Another limitation of this study is the age of the data (i.e., collected in 1995) and the extent to which these findings could be generalized to present day. Locations of destinations in established neighborhoods – from which these data were derived – are likely to be more stable than destinations located in newly developed neighborhoods. Moreover, established neighborhoods (and immediate surrounding neighborhoods) are likely to offer more recreational destination options, than newly established neighborhoods. In addition, physical activity behaviors and individual level determinants of adults in Australia have not changed dramatically in the last decade. Hence, we posit that the distances traveled to recreational destinations would be similar to those found in this study, should respondents in the same established neighborhoods be surveyed again.

## Conclusion

Spatial opportunity, destination type, trip purpose, and socio-demographic characteristics influence distances traveled to recreational destinations. Evidence from physical activity research shows that local neighborhood parks, trails, cycle paths, greenways, and beaches are visited more frequently than those located further away [15,45,49]. Therefore, it is important to know how the availability of local destinations influences frequency of use and how frequency of use influences physical activity behavior. Furthermore, simply adding more recreational facilities to neighborhoods without considering other built environmental factors (i.e., population density, connectivity) and the possible trade-offs (e.g., potential loss and fragmentation of residential land), may not have the desired affect on travel or physical activity behavior. Hence, more information about the interactions between the built environment, spatial behavior and physical activity behavior are necessary for developing neighborhoods that encourage more physical activity participation.

## Competing interests

The author(s) declare that they have no competing interests.

## Authors' contributions

GRM and BGC conceived this study. GRM collated literature, conducted the analysis and drafted the manuscript. MB assisted in the analysis and commented on drafts and BGC and TJP commented on drafts.
